# Identifying predictors of increased quantities of human Herpesvirus 8 DNA detection at oropharyngeal and plasma sites among Ugandan adults with and without HIV and Kaposi Sarcoma

**DOI:** 10.1186/1750-9378-7-S1-O23

**Published:** 2012-04-19

**Authors:** Warren Phipps, Jackson Orem, Innocent Mutyaba, James Kafeero, Meei-Li Huang, Stacy Selke, Lisa Bunts, Marla Husnik, Anna Wald, Larry Corey, Corey Casper

**Affiliations:** 1 University of Washington, Seattle, WA, USA; 2 Fred Hutchinson Cancer Research Center, Seattle, WA, USA; 3 Uganda Cancer Institute, Kampala, Uganda; 4 Makerere University, College of Health Sciences, Kampala, Uganda

## Background

Persons with KS and uncontrolled HIV infection have HHV-8 DNA detected more frequently at mucosal sites and plasma [1], but it remains unknown whether the quantity of HHV-8 detected is associated with KS development or HHV-8 transmission. We sought to characterize and determine the correlates of elevated HHV-8 DNA copy number in the oropharynx and plasma of Ugandan adults with and without HIV and KS.

## Methods

Participants collected daily oral swabs and weekly plasma samples over 4 weeks to quantify HHV-8 DNA by polymerase chain reaction.

## Results

**Figure 1:**
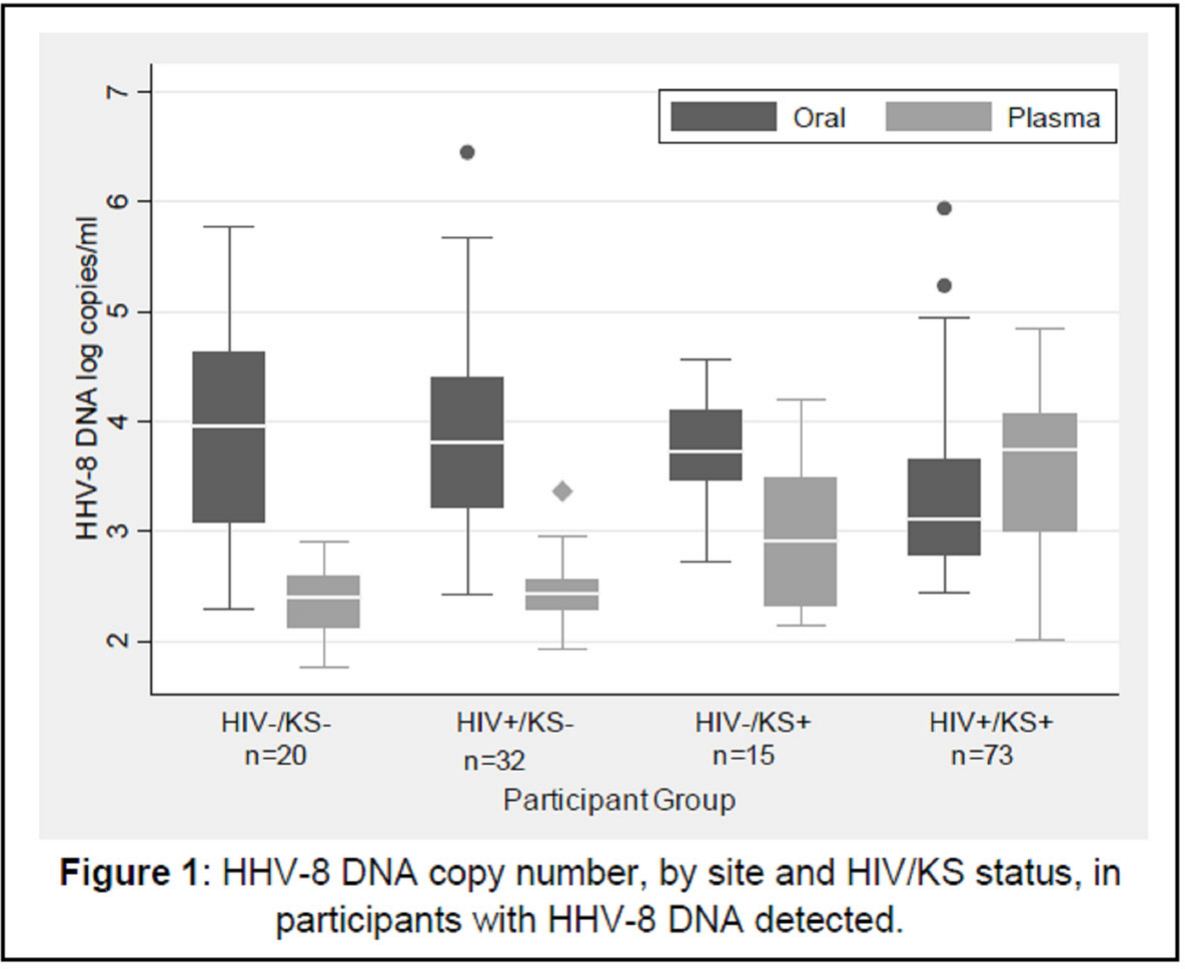


297 participants collected a total of 8,045 oral swabs and 1,392 plasma samples. HHV-8 DNA was detected in 1,561 (19%) oral swabs and 419 (30%) plasma samples. The frequency of detecting any HHV-8 differed by KS status. HHV-8 was detected in the oropharynx of 70% (64/92) persons with KS vs. 27% (52/194) without KS (p<0.001), and in the plasma of 96% (88/92) persons with KS vs. 20% (38/194) without KS (p<0.001). The median amount of HHV-8 DNA detected in oral swabs was significantly lower in persons with KS (3.2 log copies/ml) than those without KS (3.8 log copies/ml, p<0.001) (Figure 1). HHV-8 quantities in the oropharynx did not differ by participants’ HIV status (p=0.13), but elevated HHV-8 quantities were associated with CD4 counts >500 (coef 0.59, CI 0.16-1.03, p=0.007). In multivariate analysis, factors associated with higher oral HHV-8 copy number included absence of KS (coef 0.45, CI 0.14-0.75, p=0.004) and poor dentition (coef 0.37, CI 0.08-0.65, p=0.01). The median amount of HHV-8 DNA in plasma was significantly higher in persons with KS (3.6 log copies/ml) than those without KS (2.4 log copies/ml, p<0.001). In contrast to oral detection, higher plasma HHV-8 quantities were associated with CD4 counts <500 (coef 0.73, CI 0.40-1.05, p<0.001). In multivariate analysis, higher plasma HHV-8 copy number was associated with KS (coef 0.99, CI 0.80-1.17, p<0.001) and HIV infection (coef 0.39, CI 0.15-0.63, p=0.002).

## Conclusions

Increased quantities of HHV-8 DNA were detected in the oropharynx of persons without KS and those with poor dentition. The latter observation may be explained if higher CD4 counts allow for increased inflammation in the oropharynx, in turn leading to greater HHV-8 replication. Quantities of HHV-8 are higher in the plasma of persons with either HIV infection or KS, perhaps representing the propensity of HHV-8 to disseminate systemically in the absence of effective immune control or from foci of replication in KS tumors.

## References

[B1] Johnston C Orem J Okuku F Impact of HIV infection and Kaposi sarcoma on human herpesvirus-8 mucosal replication and dissemination in Uganda PLoS One 2009 4 e4222 10.1371/journal.pone.0004222 19156206 PMC2625442

